# Feasibility and Preliminary Outcomes of the Modified Makuuchi Incision in Urologic Oncology: A Single-Surgeon Case Series

**DOI:** 10.7759/cureus.108443

**Published:** 2026-05-07

**Authors:** David Nelwan, Saeed Imam, Aaron Dahmen, Timothy Nywening, Andreas Karachristos, Noah L Nellis, Trushar Patel

**Affiliations:** 1 Department of Urology, University of South Florida, Tampa, USA; 2 Department of Surgical Oncology, University of South Florida, Tampa, USA

**Keywords:** adrenal, genitourinary tumor, ivc thrombus, modified makuuchi incision, oncology, renal, renal cell carcinoma, urology

## Abstract

Introduction: The modified Makuuchi incision consists of a midline incision originating at the xiphoid process, extending caudally to the supra-umbilical region, then curving laterally as a “J/L” shape between the anterior superior iliac spine and the lowest rib. It affords not only ease of entry but also rapidity of closure, excellent exposure, and compatibility with prior abdominal incisions and any body habitus. Despite the potential benefits of the Makuuchi incision, it has had a limited role in urologic surgery. We present our experience with the utilization of the modified Makuuchi incision for large renal and adrenal tumors at our institution.

Methods: Sixteen cases utilizing the Makuuchi incision for genitourinary tumors were reviewed, all performed by a single urologist at our tertiary care institution. Data collected included patient demographics and characteristics, intraoperative metrics, and postoperative course.

Results: Our cohort consisted of seven females and nine males; 12 patients underwent right-sided incision, and four underwent left-sided incision. Twelve patients (75%) had renal cell carcinoma, and four had adrenal pathology. There was renal vein invasion/inferior vena cava thrombus in eight patients (50%). Table 1 summarizes the averages and ranges for the following data points in our 16 cases: patient age, BMI, tumor size, operative time, estimated blood loss, return of bowel function, post-anesthesia care unit oral morphine milligram equivalents (OME), and length of stay. Table 2 lists tumor size, staging, and the level of venous thrombus. There were neither intraoperative complications nor any incision-related postoperative complications.

Conclusions: The modified Makuuchi incision has been shown to be safe and feasible for large genitourinary tumors, with comparable outcomes to other incisions. Based on our cumulative operative experience, we conclude that the Makuuchi incision represents a feasible and adaptable surgical approach for complex urologic oncology cases and requires broad upper abdominal exposure.

## Introduction

Adrenal mass removal has been performed successfully using open, laparoscopic, and robotic-assisted techniques via retroperitoneal or transperitoneal approaches [1,2]. The retroperitoneal approach was the gold standard for nephrectomy until the 1950s, when effective antibiotic prophylaxis promoted the feasibility of anterior transperitoneal approaches [3].

There are several anterior transperitoneal incisions: midline, subcostal (unilateral)/Chevron (bilateral), and the Makuuchi incision [2]. Over the last 100 years, the understanding of anatomical landmarks in adrenal and kidney surgery has improved significantly; however, no single specific incision has yet been deemed categorically superior [4].

The Makuuchi incision was initially developed for enhanced right upper abdominal exposure. The modified Makuuchi incision consists of an upper midline incision extending from the xiphoid process, then curving laterally in an L or reverse L configuration parallel to the anatomic abdominal skin fold to terminate at the midpoint between the lowest rib and the anterosuperior iliac spine [5-7]. We report our institutional experience using the modified Makuuchi incision for the management of large renal and adrenal tumors.

## Materials and methods

Study population and data collection

**Figure 1:**
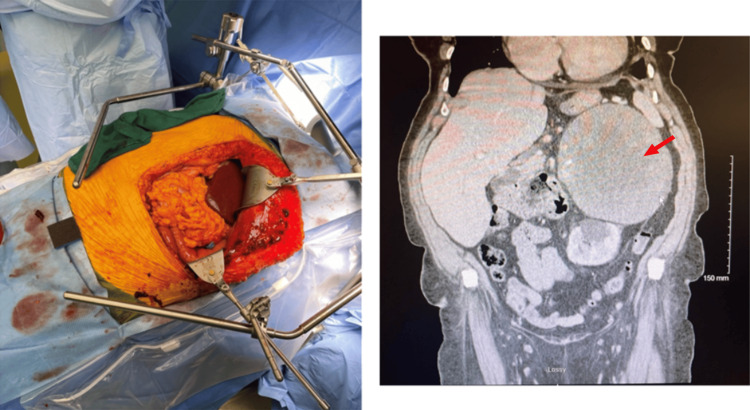
Intraoperative view of left-sided modified Makuchi incision (left); patient's CT scan showing a 13.9 cm left adrenal mass (right).

This retrospective, single-surgeon series analyzed 16 cases utilizing the modified Makuuchi incision, as described in the technical guide by Chang et al. [6], for genitourinary tumors (Figure 1) at a single tertiary-care institution. The study period spanned from September 2020 to February 2023. Adult patients (≥18 years) with a radiographic or pathologic diagnosis of a renal or adrenal tumor requiring open surgical resection were eligible for inclusion. To minimize selection bias, all cases meeting these criteria were included consecutively. Exclusion criteria included minimally invasive resections (laparoscopic or robotic), open resections performed using alternative incisions, non-oncologic resections, and cases with incomplete perioperative datasets.

Data collected included patient demographics (age, sex, and BMI), surgical characteristics (laterality and tumor size), and final pathological staging. Perioperative variables included operative time (from incision to procedure completion), estimated blood loss (EBL), and return of bowel function (defined as the time from surgery to the first passage of flatus). Postoperative metrics included length of stay (LOS), postoperative complications, and oral morphine milligram equivalents (OME), calculated from the time of discharge from the post-anesthesia care unit (PACU) through hospital discharge. Continuous data were summarized using the mean, standard deviation (SD) with Bessel's correction, and range. Data with skewed distributions are reported as median (IQR). Categorical data were expressed as frequencies. Outcomes were narratively compared with published data on the Makuuchi incision and its modifications.

Outcome measures and follow-up

For patients with venous tumor thrombus, operative time included the duration of venous thrombectomy and vascular control/reconstruction, as these components represent integral portions of the oncologic resection rather than separate procedures.

Postoperative complications were identified through retrospective review of inpatient hospitalization records, operative reports, discharge summaries, and outpatient follow-up documentation. Complications were graded according to the Clavien-Dindo classification system, which stratifies complications based on the requirement for pharmacologic therapy, procedural intervention, intensive care unit admission, or the presence of life-threatening sequelae. Follow-up duration ranged from 6 to 36 months, permitting capture of both early and delayed postoperative complications, including incisional morbidity.

Postoperative opioid utilization was quantified using OME beginning after discharge from the PACU and continuing through inpatient discharge. This timeframe was selected to exclude intraoperative anesthetic opioid administration and immediate PACU titration managed by anesthesia teams, thereby standardizing comparisons to ward-managed analgesic requirements. Focusing on post-PACU opioid use allowed assessment of patient-controlled postoperative pain management during the inpatient hospitalization period.

## Results

Our cohort consisted of seven females and nine males; 12 underwent right-sided incision, and four underwent left-sided incision. Twelve patients (75%) had renal cell carcinoma, and four had adrenal pathology. There was renal vein invasion or inferior vena cava (IVC) thrombus in eight patients (50%).

Our 16 cases had the following means ± SD (range): patient age 64.1 years ± 11.2 (40-78), BMI 29.5 ± 5.7 (21.3-37.8), tumor size 11.9 cm ± 4.0 (6.4-19), operative time 220.9 min ± 62.3 (125-338), EBL 441 cc ± 286 (100-1300), return of bowel function 4.4 days ± 0.9 (3-7). Presented as median (IQR): post-PACU OME 182 mg (99.8-259.7) and LOS five days (4-6.5) (Table 1). When stratified by presence of thrombus, the mean EBL in patients with thrombus (M = 525 cc, SD = 346) was not significantly different from patients without thrombus (M = 356 cc, SD = 197); t(14) = 1.20, p = 0.25, mean difference (95% CI) = 168.75 (-133.5, 471.0). The tumor size, staging, and level of venous thrombus for each patient are listed in Table 2. Table 3 shows a comparison of our data with those reported in the literature for the Makuuchi incision.

**Table 1:** Data expressed as mean ± SD (range) or median (IQR) for our 16 patients. PACU: post-anesthesia care unit; EBL: estimated blood loss; LOS: length of stay.

Age (years)	BMI	Tumor size (cm)	Operative time (min)	EBL (cc)	Return of bowel function (days)	Median post-PACU oral morphine equivalents (mg)	Median LOS (days)
64.1 ± 11.2 (40-78)	29.5 ± 5.7 (21.3-37.8)	11.9 ± 4.0 (6.4-19)	220.9 ± 62.3 (125-338)	441 ± 286 (100-1300)	4.4 ± 0.9 (3-7)	182 (99.8-259.7)	5 (4-6.5)

**Table 2:** Tumor staging for our 16 patients. cc: clear cell; pap: papillary; RCC: renal cell carcinoma; ACC: adrenocortical carcinoma; IVC: inferior vena cava.

Patient	Max dimension (cm)	Thrombus	Staging	Margins
1	15	-	pT3a NX ccRCC	R0
2	13	-	Adrenal myelolipoma	R0
3	13.5	Renal vein	pT3a N0 ccRCC	R0
4	16	-	pT2b N0 papRCC	R0
5	11.5	-	pT3a N0 ccRCC	R0
6	6.4	-	ACC recurrence	R0
7	15.5	-	pT2 N1 ACC	R0
8	6.5	Level II IVC	pT3b N0 ccRCC	R0
9	19	-	intra-adrenal organizing hematoma	R0
10	9.2	Renal vein	pT3a N0 ccRCC	R0
11	15	Renal vein	ypT4 ccRCC	R1 (perinephric fat, Gerota's)
12	9	Level I IVC	pT3b ccRCC	R1 (Renal vein)
13	15.5	-	pT2b N0 ccRCC	R0
14	9.9	Renal vein	pT3a N0 ccRCC	R0
15	8.4	Level II IVC	pT3a N0 ccRCC	R1 (Renal vein)
16	6.4	Level II IVC	pT3b N0 ccRCC	R1 (Renal vein)

**Table 3:** Our data points compared to those reported in existing literature for the Makuuchi incision. Data expressed as mean (range), except where listed otherwise or as denoted with *median (range). NR: not recorded; cc: clear cell; LOS: length of stay; EBL: estimated blood loss.

	Our cohort	(Ruffolo et al., 2018) [8]	(Pandit et al., 2019) [5]	(Polat et al., 2019) [9]	(Bokka et al., 2010) [7]
# of cases	16 (12 renal, 4 adrenal)	41 adrenal	144 (upper abdominal)	29 renal	18 (15 renal, 3 adrenal)
Incision	modified Makuuchi	Makuuchi	modified Makuuchi	modified Makuuchi	modified Makuuchi
Age (years)	64.1 (40-78)	51.7 (19-86)	48.3 (8-78)	58.5 (48-72)	52 (±12.2)
BMI	29.5 (21.3-37.8)	29.7 (17.3-45.8)	NR	NR	NR
Tumor size (cm)	11.9 (6.4-19)	8 (3.1-26)	NR	11.3 (5-16)	13.8 (±6.3)
Operative time (min)	220.9 (125-338)	333 (186-577)*	NR	NR	369.7 (±210.6)
EBL (cc)	441 (100-1300)	NR	NR	NR	1123.5 (±990.3)
LOS (days)	7.2 (3-19)	6 (4-73)*	NR	NR	11.65 (±13.2)
Incision-related postoperative complications	0	5 hernias, 3 surgical site infections	19 surgical site infections, 6 hernias	0	1 surgical site infection
Follow-up (months)	NR	median: 16	mean: 28	NR	NR

There were no intraoperative complications, surgical site infections, incisional hernias, or other incision-related complications identified during the follow-up period (range: 6-36 months). One patient was returned to the operating room for concern of mesenteric ischemia or superior mesenteric artery thrombosis; the subsequent exploratory laparotomy was negative, representing a Clavien-Dindo Grade IIIb event. Two patients who underwent IVC thrombectomy experienced prolonged postoperative hospital stays of 19 days each due to medical complications: one sustained a postoperative myocardial infarction, and the other developed postoperative day two hypotension and hypoxia necessitating intubation and continuous renal replacement therapy (CRRT), both classified as Clavien-Dindo Grade IV events.

## Discussion

Historically, the extraperitoneal lumbar approach for nephrectomy was preferred due to perceived safety [10-11]. The advent of effective antibiotic prophylaxis in the mid-20th century led to decreased rates of peritonitis and a consequent increase in operations via a transperitoneal approach [3,12-15].

In 1993, Masatoshi Makuuchi developed the “Makuuchi incision” for enhanced right upper quadrant access: a midline incision originating at the xiphoid process, extending caudally to the supra-umbilical region, then curving laterally as a "J/L" shape along the 9th intercostal space and ending at an axillary line [5-7]. Exposure is obtained by folding the rectus abdominis musculocutaneous flap superolaterally, and it can be fixed in place with the use of sutures or retractors [7]. Successful usage of the Makuuchi incision has been described for a variety of major upper abdominal surgeries, including Whipple’s pancreaticoduodenectomy, hepatectomy, cholecystectomy, gastrectomy, hemicolectomy, splenorenal shunt, and hepatic artery pseudoaneurysm repair [5]. Ruffolo et al. (2018) retrospectively reviewed 41 open adrenalectomies using the Makuuchi incision, with a mean tumor diameter of 8 cm (3.1-26), a median operative time of 333 min (186-577), and a median LOS of 6 days (4-73). They concluded that the Makuuchi incision was well-tolerated, provided exceptional exposure, and allowed for ideal adrenal access [8].

The modified Makuuchi incision, described by Chang et al. in 2010, curves laterally between the anterior superior iliac spine and the lowest rib, obviating the division of intercostal muscles and leading to reduced postoperative pain [5-6]. Pandit et al. (2019) reported on 144 modified Makuuchi cases done at three hospitals in India for major upper abdominal surgeries, highlighting the optimal exposure provided by the modified Makuuchi incision, as well as ease of extension with other incisions if necessary, ability to use on any body habitus, and ease of usage with previous incisions. They emphasized that the Modified Makuuchi incision preserves abdominal wall integrity with comparable surgical site infection rates and good pain control [5].

The Makuuchi incision eventually garnered attention from urologists due to its excellent exposure/access to the kidneys, adrenals, great vessels, and renal pedicles. Polat et al. (2019) utilized the modified Makuuchi incision in 29 renal tumor cases (26 radical nephrectomies and three partial nephrectomies). The average tumor size was 11.3 cm (5-16) in their radical nephrectomy cohort, 11 of whom had venous thrombi up to level II. There were no reported incisional hernias, infections, or other complications, and there was no increased analgesic use compared to other incisions for renal surgery. Once again, the unparalleled exposure was lauded by the authors, particularly in the setting of comparable outcomes in other postoperative metrics like pain control, complications, and cosmesis [9].

Bokka et al. (2020) further expanded on the urologic success of the modified Makuuchi incision with 18 renal and adrenal cases at a single institution in India. Their cohort included two radical nephrectomies with non-segmental liver resection and seven radical nephrectomies with IVC thrombectomies (including one level IV thrombus and two level III thrombi). Mean tumor size was 13.8 cm (±6.3), mean operative time was 369.7 min (±210.6), mean EBL was 1123.5 cc (±990.3), and mean hospital stay was 11.65 days (±13.2). They had one readmission for surgical site infection, and there were no reported incisional hernias. The authors concluded that the excellent exposure of the modified Makuuchi is particularly ideal for large and/or complex procedures, especially if multiple surgical specialties are involved [7].

With comparable tumor size, our modified Makuuchi cohort demonstrated a mean operative time of 220.9 min (125-338), EBL of 441 cc (100-1300), and an LOS of 7.2 days (3-19), which compare favorably with the existing literature on urologic utilization of the modified Makuuchi incision, as can be seen in Table 3. Furthermore, our cohort has not had any surgical site infections, incisional hernias, or other incision-related complications during the period of follow-up (range 6-36 months). A review of case series in the literature describing other oncologic incisions in urology also showed comparable EBL (averages from 150 to 1237 cc for the thoracoabdominal approach, 209 to 364 cc for open flank, and 103 to 400 for mini-flank) and mean LOS (4.2-9.5 days for thoracoabdominal, 4.0-9.4 for open flank, and 5.0-6.8 for mini-flank) [16]. Although no intraoperative or incision-related complications were observed, two surgical outcomes warrant further discussion: R1 resection margins in four patients (25%) and two Clavien-Dindo Grade IV complications.

The 25% positive margin rate in our study reflects the inherent technical challenges of radical nephrectomy with IVC thrombectomy. The IVC lumen represents a confined and partially “blind” surgical field, limiting direct visualization of proximal and distal margins during thrombus extraction. Residual tumor adherent to the IVC wall or microscopic wall invasion may not be apparent intraoperatively, and preoperative imaging cannot reliably distinguish thrombus from true vascular invasion. Higher-level thrombi further increase complexity due to the need for extensive exposure, vascular control, and hemodynamic management, which constrain meticulous margin assessment. Reported positive vascular margin rates of 18-23% [17,18] in existing literature underscore that our findings are consistent with the known technical difficulty of these cases rather than inadequate oncologic resection. This rate is oncologically significant; however, as positive vascular margins were found to be predictive of local recurrence, they are not predictive of systemic recurrence or cancer specific survival [17].

Grade IV (life-threatening) complications occur in approximately 16-18% of patients undergoing IVC thrombectomy, with risk strongly influenced by thrombus level and institutional experience [19]. Henning et al. (2025) found that national and multicenter data demonstrate substantially higher complication rates in patients with levels III-IV (suprahepatic or supradiaphragmatic) thrombi, where cardiopulmonary and hematologic events predominate and major complication rates may exceed 40% in the most advanced cases. Hospital volume is a critical determinant of outcomes, with high-volume centers demonstrating significantly lower major complication rates compared to low-volume institutions [19]. Our institution is a high-volume referral center, which is an important factor in mitigating perioperative risk.

Finally, several limitations to our study must be acknowledged. As a retrospective, non-randomized, single-surgeon and single-center analysis, it is inherently subject to selection and performance bias, which may limit generalizability. The decision to use the Makuuchi incision was at the surgeon's discretion, introducing selection bias that consecutive inclusion cannot fully mitigate. Outcomes may reflect the specific patient volume and expertise of our surgical team rather than the inherent advantages of the incision itself. The relatively short follow-up period restricts interpretation of long-term survival and oncologic outcomes. Additionally, the absence of quality of life data precludes assessment of postoperative functional recovery and patient comfort compared with other incisions. Furthermore, cross-series comparisons are subject to significant methodological limitations. Differences in patient selection, case complexity, surgeon experience, and anesthetic protocols between studies may confound these results. Prospective comparative studies are warranted to more rigorously evaluate these outcomes.

## Conclusions

Based on our cumulative operative experience, the modified Makuuchi incision allows for broad mobilization and retraction of the abdominal wall flap, providing enhanced access to both the ipsilateral and contralateral abdominal quadrants. This improved exposure facilitates mobilization of adjacent structures, including the spleen and liver, which can be particularly advantageous for the management of large and complex renal and adrenal tumors, including cases with venous involvement. In our series, perioperative outcomes, including LOS, EBL, and complications, were comparable to existing literature on urologic utilization of the modified Makuuchi incision.

The modified Makuuchi incision has been shown to be safe and feasible for large genitourinary tumors, with comparable outcomes to other incisions. As open surgery continues to play a critical role in select patients with advanced or anatomically complex genitourinary malignancies, this incision could represent a valuable option within the urologic surgeon’s operative repertoire.
